# Missing-in-metastasis B (MIM-B) combined with caveolin-1 promotes metastasis of hepatocellular carcinoma

**DOI:** 10.18632/oncotarget.20735

**Published:** 2017-09-08

**Authors:** Xiu-Yan Huang, Zi-Li Huang, Tao Niu, Zhen-Qian Wu, Bin Xu, Yong-Hua Xu, Xin-Yu Huang, Qi Zheng, Jian Zhou, Zi Chen, Zhao-You Tang

**Affiliations:** ^1^ Department of General Surgery, Shanghai Jiaotong University Affiliated Sixth People's Hospital, Shanghai, P.R. China; ^2^ Department of Radiology, Xuhui Central Hospital, Shanghai, P.R. China; ^3^ Department of General Surgery, People's Hospital of Menghai County, Yunnan Province, P.R. China; ^4^ Department of General Surgery, The Tenth People's Hospital of Tongji University, Shanghai, P.R. China; ^5^ Liver Cancer Institute and Zhongshan Hospital, Fudan University, Shanghai, P.R. China; ^6^ Thayer School of Engineering, Norris Cotton Cancer Center, Dartmouth College, Hanover, NH, USA

**Keywords:** hepatocellular carcinoma, missing in metastasis B, caveolin-1, epidermal growth factor receptor, metastasis

## Abstract

**Background:**

Increasing amounts of evidence indicate that Missing in metastasis B (MIM-B) promotes cancer metastasis. Here, we sought to better understand the mechanism through which MIM-B promotes tumor metastasis in hepatocellular carcinoma (HCC).

**Methods:**

We performed confocal microscopy analysis to determine the distributions of MIM-B and caveolin-1 and conducted co-immunoprecipitation assays to detect the interactions between MIM-B and caveolin-1 *in vitro*. We performed transwell assays to analyze the invasive ability of HCC cells. Changes in the expression levels of key genes and some molecular makers were detected by immunohistochemistry and western blotting in HCC tissue samples.

**Results:**

We found that MIM-B co-localizes with caveolin-1 and demonstrated that MIM-B and caveolin-1 interact *in vitro*. Repressing MIM-B and caveolin-1 expression inhibited the epidermal growth factor receptor signaling pathway. We overexpressed MIM-B and caveolin-1 in Hep3B cells, which enhanced Hep3B cell invasiveness. Furthermore, MHCC97H cell invasiveness was significantly decreased in cells in which MIM-B and caveolin-1 expression was inhibited. Additionally, we found that MIM-B and caveolin-1 were expressed at higher levels in HCC tissues than in paired normal tissues. Moreover, HCC patients with MIM-B and caveolin-1 up-regulation experienced significantly worse outcomes than controls (*P* < 0.001), and HCC patients with high MIM-B and caveolin-1 expression levels often developed pulmonary metastasis (*P* < 0.001).

**Conclusions:**

MIM-B combined with caveolin-1 promotes metastasis of HCC, and elevated MIM-B and caveolin-1 expression levels are associated with a poor prognosis in HCC patients; therefore, MIM-B and caveolin-1 may represent novel targets for the diagnosis and treatment of HCC.

## INTRODUCTION

Primary liver cancer is one of most commonly diagnosed cancers and is also one of the leading causes of cancer-related death worldwide. Hepatocellular carcinoma (HCC) is the most common type of primary liver cancer and accounts for approximately 75% of liver tumors [1]. Hepatic resection is currently the most frequently used treatment for patients with HCC [2]. However, HCC is usually diagnosed at an advanced stage, which limits the effectiveness of this treatment. Thus, the rates of HCC recurrence and metastasis are very high at 5 years after surgery, even among patients who have undergone radical hepatic resection [3]. Therefore, the post-resection biological features of HCC warrant further study.

Metastasis suppressor 1 (MIM-B, also known as missing in metastasis B) was found to be a potential metastasis suppressor in bladder cancer [4, 5]. The MIM gene encodes a 5.3-kb mRNA molecule, and the following three alternatively spliced isoforms of MIM are found in humans: MIM-A, MIM-B and MIM-C. MIM-B is the most common type of MIM [6]. Several studies have reported that MIM-B expression is altered in a variety of tumors, such as bladder, prostate, colorectal, ovarian, breast, neck, and gastric tumors; basal cell carcinoma; and non-small cell lung cancer, as well as HCC [4–14]. However, the role of MIM-B in different cancers seems to be a subject of controversy, as some reports have proposed that MIM-B is a potential metastasis suppressor, while others have shown that MIM-B may promote tumor metastasis [12, 14–18]. MIM-B contains multiple functional motifs, including an N-terminal IRSp53/MIM domain motif (IMD) and a C-terminal Wiskott-Aldrich syndrome protein homology 2 motif (WH2) [19]. Because of these functional motifs, MIM-B is also postulated to be an important regulator of the actincytoskeleton [20–22].

Caveolin-1, a 21–24-kDa membrane protein, is a member of the caveolin family of proteins and is also the main structural component of caveolae [23–25]. Caveolin-1 plays key roles in several cellular processes, such as caveolae-mediated vesicular transport and endocytosis, lipid metabolism, cell adhesion, cell migration, cell signaling platform regulation, cell transformation, cell proliferation, cell cycle arrest, anchorage-independent growth and apoptotic cell death [26, 27]. Recent reports showed that *caveolin-1* is a candidate tumor suppressor gene and participates in the pathogenesis of oncogenic cell transformation and tumorigenesis. Moreover, several reports have documented that caveolin-1 expression is altered in various cancers, such as bladder, ovarian, thyroid follicular, breast, esophageal, lung, colon, cervical, and renal cancer; T cell leukemia; and HCC [25, 27–31]. Notably, caveolin-1 has different functions in variety of cancers and may act both as a tumor suppressor and as a tumor-promoting gene. In recent years, several *in vitro* and *in vivo* studies have demonstrated that highly expressed caveolin-1 plays an important role in tumor cell survival and tumor growth, tumor aggressiveness, tumor metastatic potential, and tumor apoptosis and is associated with enhanced metastatic potential and poorer patient prognoses [31–35]. However, little is known about the molecular mechanisms governing the function of caveolin-1 in HCC.

The epidermal growth factor receptor (EGFR, also known as ErbB1), a 170-kDa transmembrane tyrosine kinase receptor, is a member of the receptor tyrosine kinases family and promotes cell proliferation, migration, and differentiation in variety of cancers [36–40]. Previous studies have demonstrated that MIM-B can activate the EGFR pathway in Drosophila oocyte border cell migration and endocytosis [41], and another work reported that MIM-B not only promoted EGFR expression in early stage HNSCC cells/tumors but also inhibited EGFR signaling at high cell densities [20, 42]. Moreover, previous studies have demonstrated that the role of caveolae in endocytosis was independent of the EGFR internalization pathway [43, 44] and that caveolin-1 can be co-localized with the endosomal EGFR and act upon internalized EGF endosomes [28, 45, 46]. Therefore, we are interested in determining the roles of MIM-B and caveolin-1 in the regulation of EGFR signaling.

In this study, we sought to better understand how MIM-B regulates tumor metastasis. We demonstrated that MIM-B and caveolin-1 co-localize in HCC. We also verified that MIM-B and caveolin-1 interact *in vitro*. Moreover, we investigated the relationships between MIM-B and caveolin-1 and EGFR signaling and found that inhibiting MIM-B and caveolin-1 repressed EGFR pathway activity. In addition, we demonstrated that repression in MIM-B and caveolin-1 expression levels could inhibit MHCC97H cell migration. Furthermore, we showed that MIM-B and caveolin-1 were expressed at higher levels in cancer tissues than in paired adjacent normal tissues and that MIM-B and caveolin-1 expression levels were correlated with HCC clinico-pathologic characteristics. We also showed that patients with HCC with MIM-B and caveolin-1 up-regulation experienced significantly worse outcomes and had lower survival rates than patients with HCC without MIM-B and caveolin-1 up-regulation. Thus, the interactions between MIM-B and caveolin-1 play an important role in HCC cell invasion.

## RESULTS

### MIM-B interacted with caveolin-1 *in vitro*

**Figure 1:**
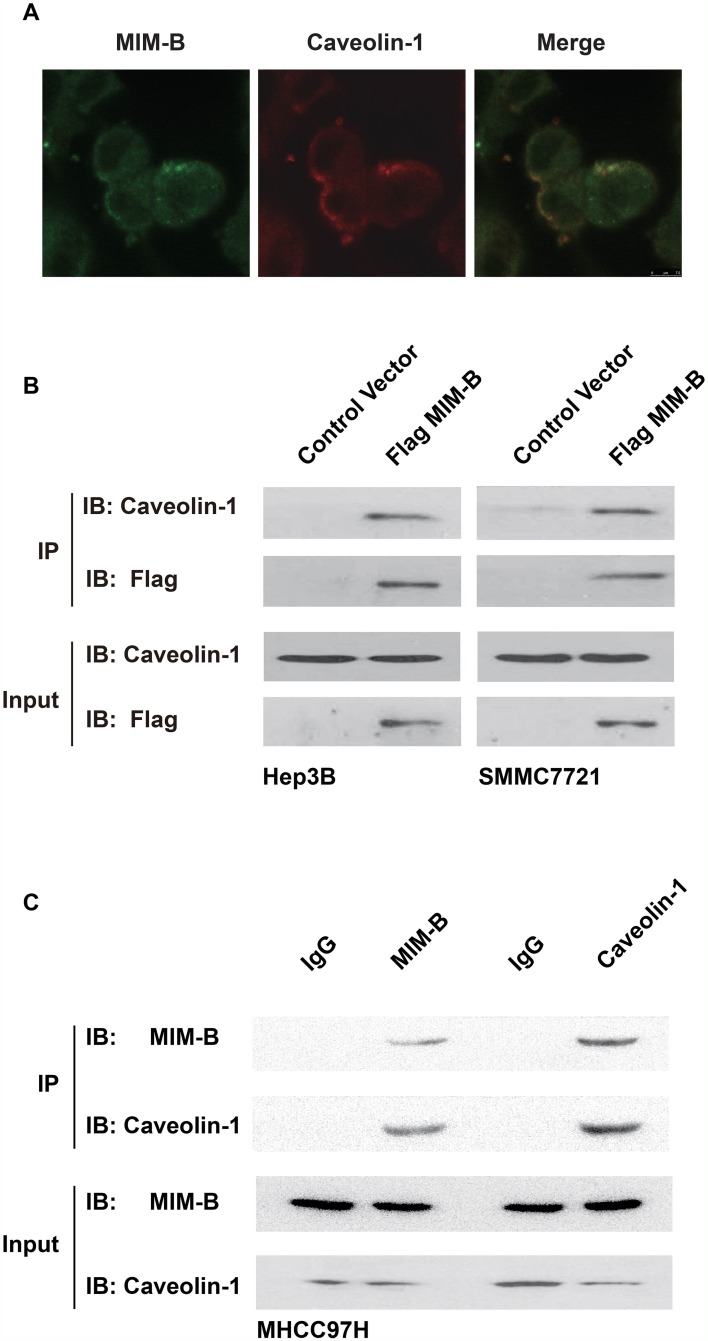
MIM-B interacted with caveolin-1 *in vitro* **(A)** The distributions of MIM-B and caveolin-1 in HMCC97H cells were analyzed by confocal microscopy. Scalebar, 10 μm. **(B)** Co-IP assay of the interactions between MIM-B and caveolin-1 *in vitro*. As shown on the left and right, anti-Flag antibodies were used for immunoprecipitation, and anti-caveolin-1 antibodies were used for immunoblotting in Hep3B and SMMC7721 cells, respectively. **(C)** Co-IP assay of the interactions between MIM-B and caveolin-1 *in vitro*. As shown on the left and right, anti- MIM-B and anti-caveolin-1 affinity matrices were used for immunoprecipitation, respectively. In this experiment, the input samples were used as controls. Each experiment was repeated a minimum of three times.

We performed cell immunofluorescence assays to detect the distributions of MIM-B and caveolin-1 in MHCC97H cells. The results, as demonstrated by confocal microscopy, showed that MIM-B and caveolin-1 are co-localized in MHCC97H cells (Figure 1A).

Given that caveolin-1 is co-localized with MIM-B, we proposed that MIM-B interacted with caveolin-1. To test this hypothesis, we performed Co-IP assays to confirm that MIM-B interacted with caveolin-1 *in vitro*. We transfected Flag-tagged MIM-B into the indicated cells and examined the interactions between MIM-B and caveolin-1. The results showed that endogenous caveolin-1 interacted with Flag-MIM-B (Figure 1B). Furthermore, we performed Co-IP experiments in MHCC97H cells. As expected, we found that anti-human MIM-B polyclonal antibodies could precipitate endogenous caveolin-1 protein and that anti-human caveolin-1 polyclonal antibodies could precipitate endogenous MIM-B protein in MHCC97H cells (Figure 1C). Taken together, these results demonstrated that MIM-B interacts directly with caveolin-1 in HCC cell lines.

### The IMD and WH2 domains of MIM-B are essential for the interactions between MIM-B and caveolin-1

**Figure 2:**
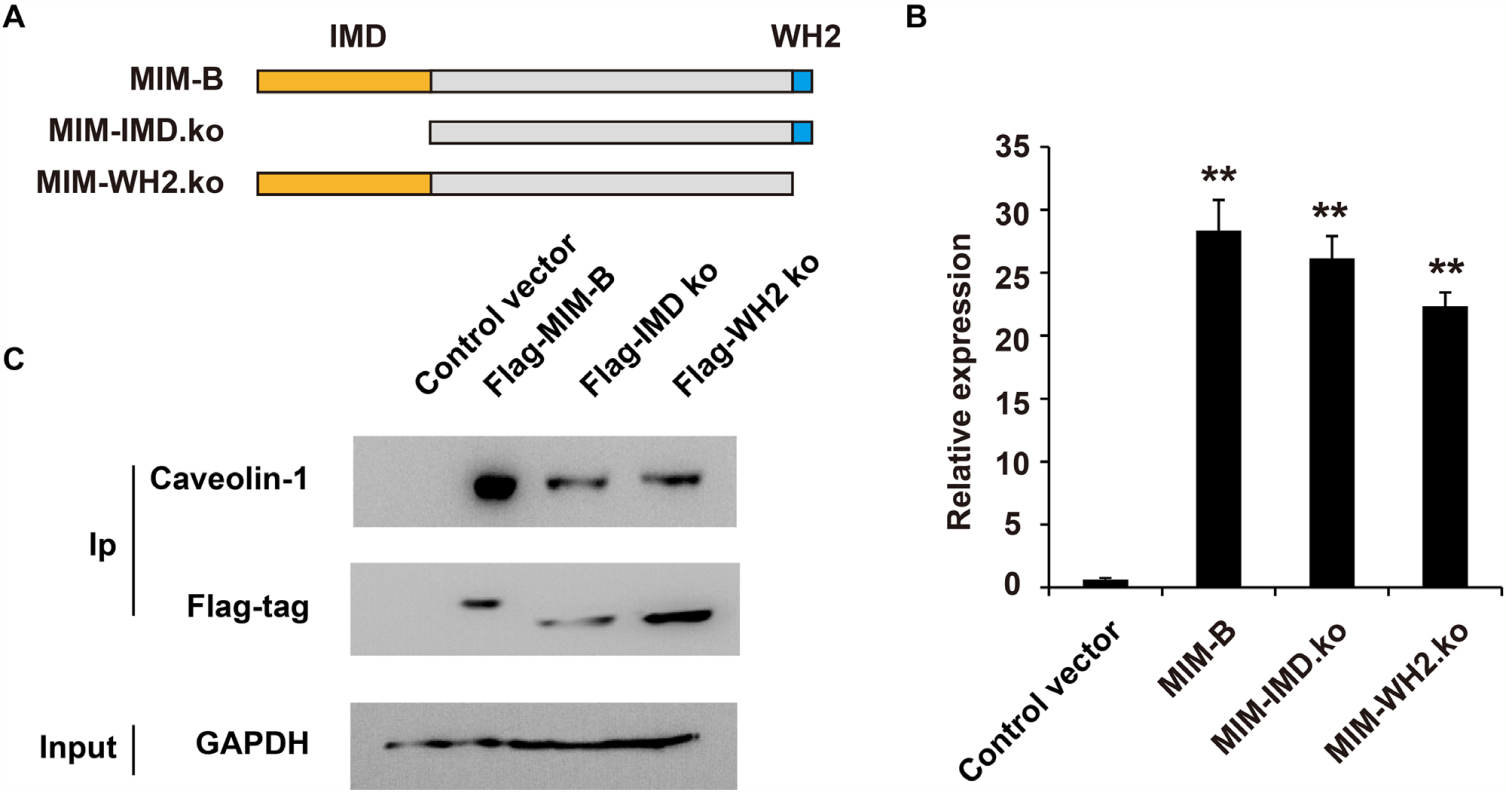
The IMD and WH2 domains are essential for the interactions between MIM-B and caveolin-1 **(A)** Diagram showing the construction of the mutant MIM-B plasmid. **(B)** RT-qPCR analysis of the mutant in transaction in HEK293 cells. **(C)** Co-IP assay of the influence of the IMD and WH2 motifs on the interaction between MIM-B and caveolin-1. In this experiment, the input samples were used as controls. ^*^P < 0.05, ^**^P < 0.01, and each experiment was repeated at least three times.

Recent reports have shown that the IMD and WH2 motifs of MIM-B may play a role in the interactions between MIM-B and other proteins to modulate cellular processes, such as cancer metastasis and cytoskeletal dynamics [15, 47]. Therefore, we constructed MIM-B sequences lacking IMD or WH2 and studied the roles of each motif in the interactions between MIM-B and caveolin-1 (Figure 2A). qRT-PCR showed that the truncated proteins were expressed successfully in HEK293 cells (Figure 2B), and the Co-IP results showed that deletion of either IMD or WH2 decreased the interactions between MIM-B and caveolin-1 (Figure 2C). These results demonstrated that the IMD and WH2 motifs are important for the interactions between MIM-B and caveolin-1.

Taken together, these results showed that MIM-B could interact with caveolin-1 *in vitro* and that the IMD and WH2 motifs of MIM-B are required for the interactions between the two proteins.

### Inhibition of MIM-B and caveolin-1 repressed EGFR pathway activity

**Figure 3:**
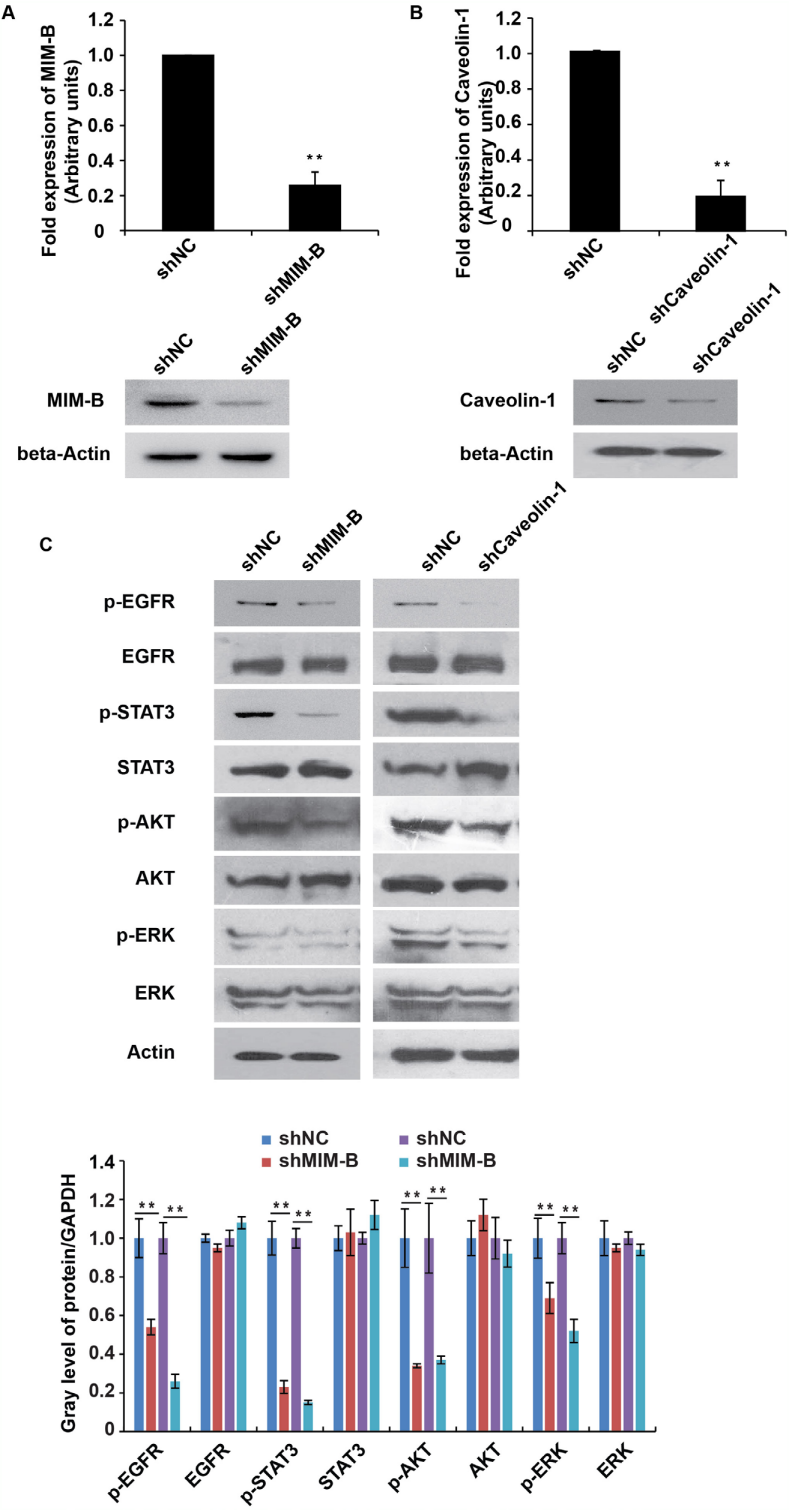
Inhibition of MIM-B and caveolin-1 repressed EGFR pathway activity **(A)** qRT-PCR and western blot analyses of MIM-B expression in MHCC97H cells transfected with shRNAs against MIM-B for approximately 48 h. **(B)** qRT-PCR and western blot analyses of caveolin-1 expression in MHCC97H cells transfected with shRNAs against caveolin-1 for approximately 48 h. **(C)** The expression levels of EGFR, STAT3, AKT and ERK and their phosphorylated counterparts were examined in cells transfected with shRNA-MIM-B and caveolin-1 for approximately 48 h. ^*^P < 0.05, ^**^P < 0.01, and each experiment was repeated at least three times.

Given that caveolin-1 interacted with MIM-B, we tested whether caveolin-1 can modulate EGFR pathway activity. We screened for shRNAs that can knock down MIM-B and caveolin-1 expression in MHCC97H cells using lentiviruses. qPCR analysis showed that shRNA against MIM-B or caveolin-1 could inhibit MIM-B (Figure 3A) or caveolin-1 expression (Figure 3B), respectively.

To elucidate the possible effects of MIM-B and caveolin-1 on the EGFR pathway, we tested the protein expression levels and phosphorylation statuses of the following four well-characterized EGFR subfamily proteins: EGFR, STAT3, AKT and ERK. Interestingly, we found that p-EGFR, p-STAT3, p-AKT and p-ERK expression levels were significantly decreased in MHCC97H cells when MIM-B and caveolin-1 were knocked down (Figure 3C).

Taken together, these results demonstrated that both MIM-B and caveolin-1 stimulated the EGFR pathway.

### Alterations in MIM-B and caveolin-1 expression levels affect HCC cell migration

**Figure 4:**
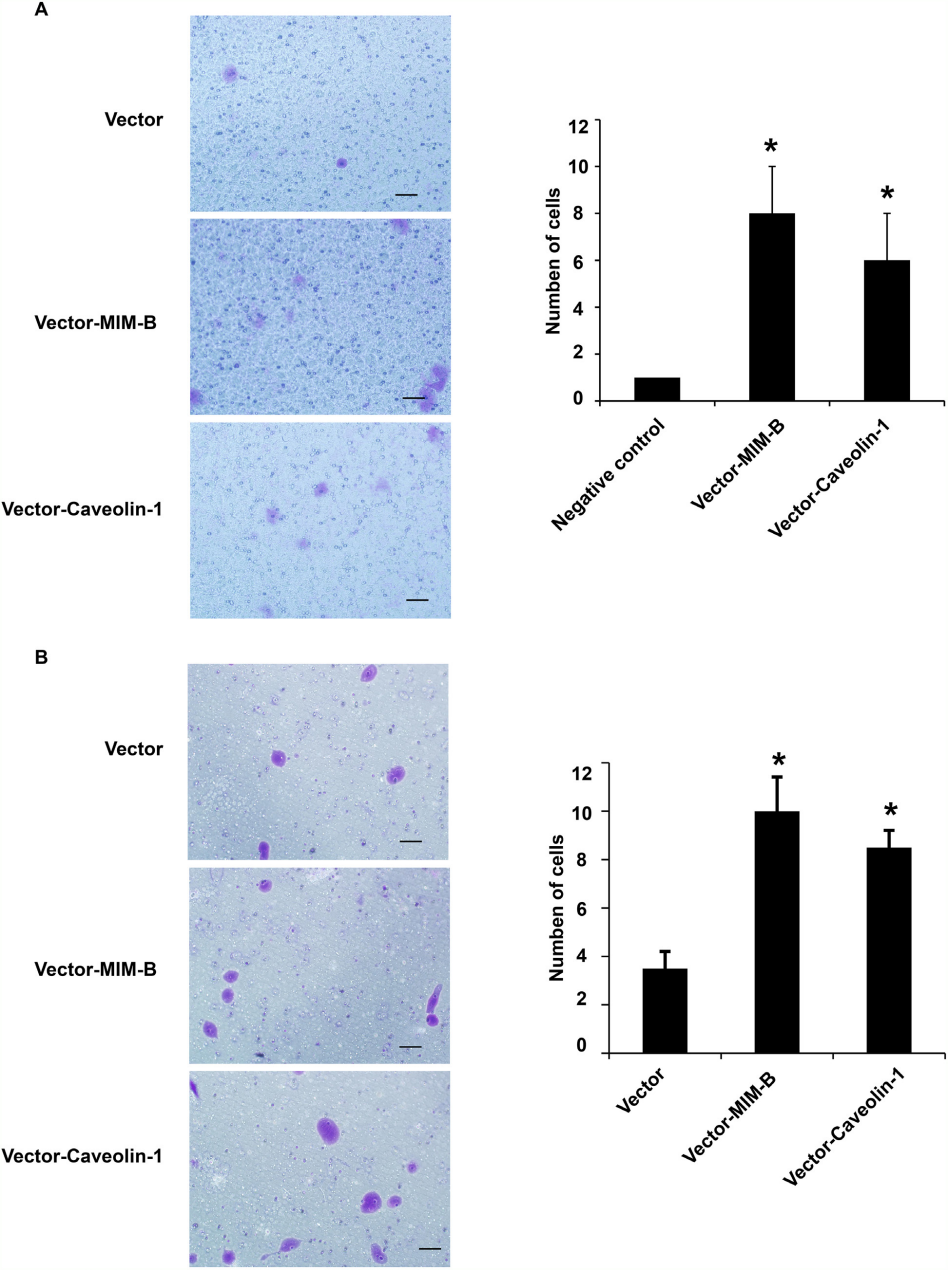
MIM-B and caveolin-1 overexpression promoted Hep3B cell migration **(A)** Transwell migration assay of Hep3B cells transfected with pcDNA-MIM-B and pcDNA-caveolin-1 plasmids. **(B)** Transwell invasion assay of Hep3B cells transfected with pcDNA-MIM-B and pcDNA-caveolin-1 plasmids. ^*^P < 0.05, ^**^P < 0.01, and each experiment was repeated at least three times.

**Figure 5:**
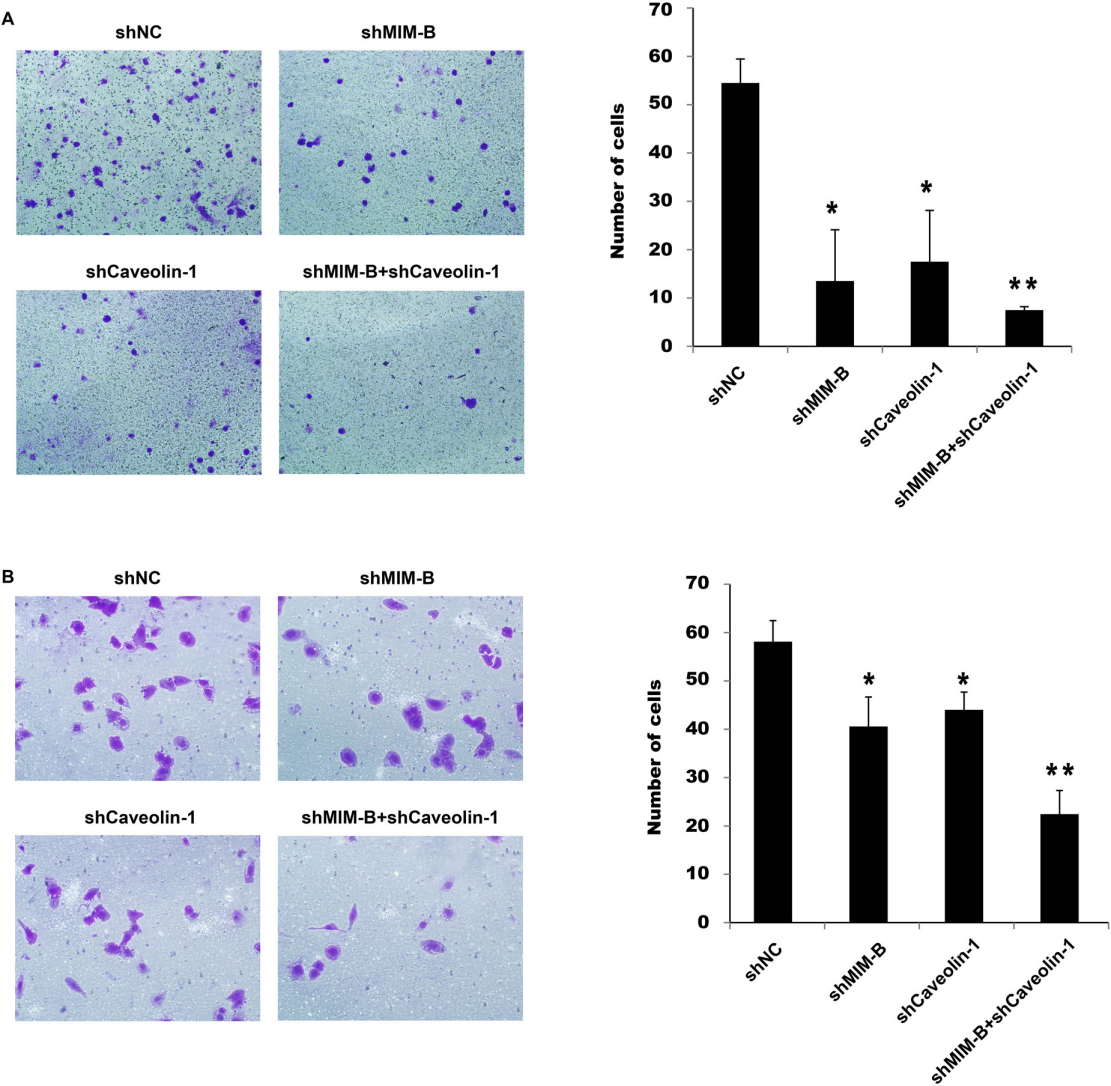
Knocking down MIM-B and caveolin-1 inhibited MHCC97H cell migration and invasion **(A)** Transwell migration assay of MHCC97H cells transfected with shMIM-B and shcaveolin-1. **(B)** Transwell invasion assay of MHCC97H cells transfected with shMIM-B and shcaveolin-1. ^*^P < 0.05, ^**^P < 0.01, and each experiment was repeated at least three times.

Given that MIM-B interacts with caveolin-1 in HCC cells, we investigated whether MIM-B and caveolin-1 affect HCC cell invasion and metastasis. We over-expressed MIM-B and caveolin-1 in Hep3B cells and performed cell migration assay, the results of which showed that Hep3B cell metastasis was significantly increased in the corresponding cells compared with control cells (Figure 4A and 4B), demonstrating that MIM-B and caveolin-1 could promote Hep3B cell migration. Furthermore, we downregulated MIM-B expression and found that MHCC97H cell migration (Figure 5A, lanes 1 and 2) and MHCC97H cell invasion (Figure 5B, lanes 1 and 2) were decreased in the corresponding cells compared with control cells. These results demonstrated that MIM-B positively promoted MHCC97H cell invasion. To determine the role of caveolin-1 in MHCC97H cell invasion, we reduced caveolin-1 expression and found that MHCC97H cell metastasis was inhibited in the corresponding cells compared with control cells (Figure 5A and 5B, lanes 1 and 3). These results showed that caveolin-1 enhances MHCC97H cell invasiveness.

To determine the importance of MIM-B and caveolin-1 in MHCC97H cell invasiveness, we knocked down MIM-B and caveolin-1 expression simultaneously, and found that MHCC97H cell migration and invasiveness were significantly decreased in the corresponding cells compared with control cells (Figure 5, lanes 1 and 4).

Taken together, these results indicate that altering MIM-B and caveolin-1 expression affected HCC cell migration.

### Correlation between MIM-B and caveolin-1 expression in HCC

To determine the clinical significance of MIM-B and caveolin-1 expression in HCC, we examined MIM-B and caveolin-1 expression in HCC tissues. The correlations between MIM-B and caveolin-1 expression levels, as determined by IHC, and HCC clinico-pathologic characteristics of HCC are summarized in Table 1. The majority of patients with HCC had high MIM-B (n = 49, 49/84, 58.3%) or caveolin-1 expression levels (n = 53, 53/84, 63.1%). Twenty-five (25/38, 65.8%) of 38 cases of HCC with a tumor size < 5 cm presented with high MIM-B expression, while 24 (24/46, 52.2%) of 46 cases of HCC with a tumor size ≥ 5 cm presented with high MIM-B expression. High MIM-B and caveolin-1 expression was correlated with tumor satellites (*P* = 0.005), numbers (*P* = 0.006), encapsulation (*P* = 0.027), vascular invasion (*P* = 0.001) and TNM stage (*P* = 0.002). We also observed that pulmonary metastasis was more common in HCC with high MIM-B expression levels (28/40, 70%) than in HCC with lower MIM-B expression levels (12/40, 30%; *P* = 0.039) and in HCC with high caveolin-1 expression levels (32/40, 80%) than in HCC with lowers caveolin-1 expression levels (8/40, 20%; *P* = 0.002). Furthermore, the majority of patients with pulmonary metastasis (27/38, 71.1%; *P* < 0.001) had both high MIM-B expression and high caveolin-1 expression. Taken together, these data demonstrated that MIM-B and caveolin-1 levels were associated with HCC clinico-pathologic characteristics.

**Table 1:** Correlations between MIM-B and caveolin-1 expression and the clinicopathological parameters of patients with HCC

Features		No.	MIM-B expression		*P*	Caveolin-1 expression		*P*	MIM-B and caveolin-1 expression		*P*
			High	Low		High	Low		Both high	None	
**Gender**	Male	73	42	31	0.702	45	28	0.478	33	40	0.988
	Female	11	7	4		8	3		5	6	
**Age**	< 60 y	63	34	29	0.160	40	23	0.896	30	33	0.448
	≥ 60 y	21	15	6		13	8		8	13	
**AFP level, ng/ml**	≤ 20	26	14	12	0.576	13	13	0.096	10	16	0.403
	> 20	58	35	23		40	18		28	30	
**Cirrhosis**	Yes	60	38	22	0.142	35	25	0.153	31	29	0.061
	No	24	11	13		18	6		7	17	
**Satellite**	Yes	21	17	4	0.021	18	3	0.018	15	6	0.005
	No	63	32	31		35	28		23	40	
**Tumor number**	Multiple	46	35	11	0.000	34	12	0.024	27	19	0.006
	Single	38	14	24		19	19		11	27	
**Encapsulation**	Complete	33	13	20	0.005	15	18	0.007	10	23	0.027
	Incomplete	51	36	15		38	13		28	23	
**Vascular invasion**	Yes	53	41	12	0.000	39	14	0.009	31	22	0.001
	No	31	8	23		14	17		7	24	
**Tumor size**	< 5cm	38	25	13	0.208	26	12	0.358	16	22	0.600
	≥ 5cm	46	24	22		27	19		22	24	
**Disease stage (TNM)**	I	10	7	3	0.032	6	4	0.016	4	6	0.002
	II	25	11	14		11	14		5	20	
	III	33	17	16		21	12		16	17	
	IVA	16	14	2		15	1		13	3	
**Tumor stage**	T1	12	9	3		8	4		5	7	0.379
	T2	33	22	11		25	8		18	15	
	T3	39	18	21		20	19		15	24	

NOTE: HCC, hepatocellular carcinoma. No patients had T4 or IVB disease in this study. Significant difference: P < 0.05.

**Figure 6:**
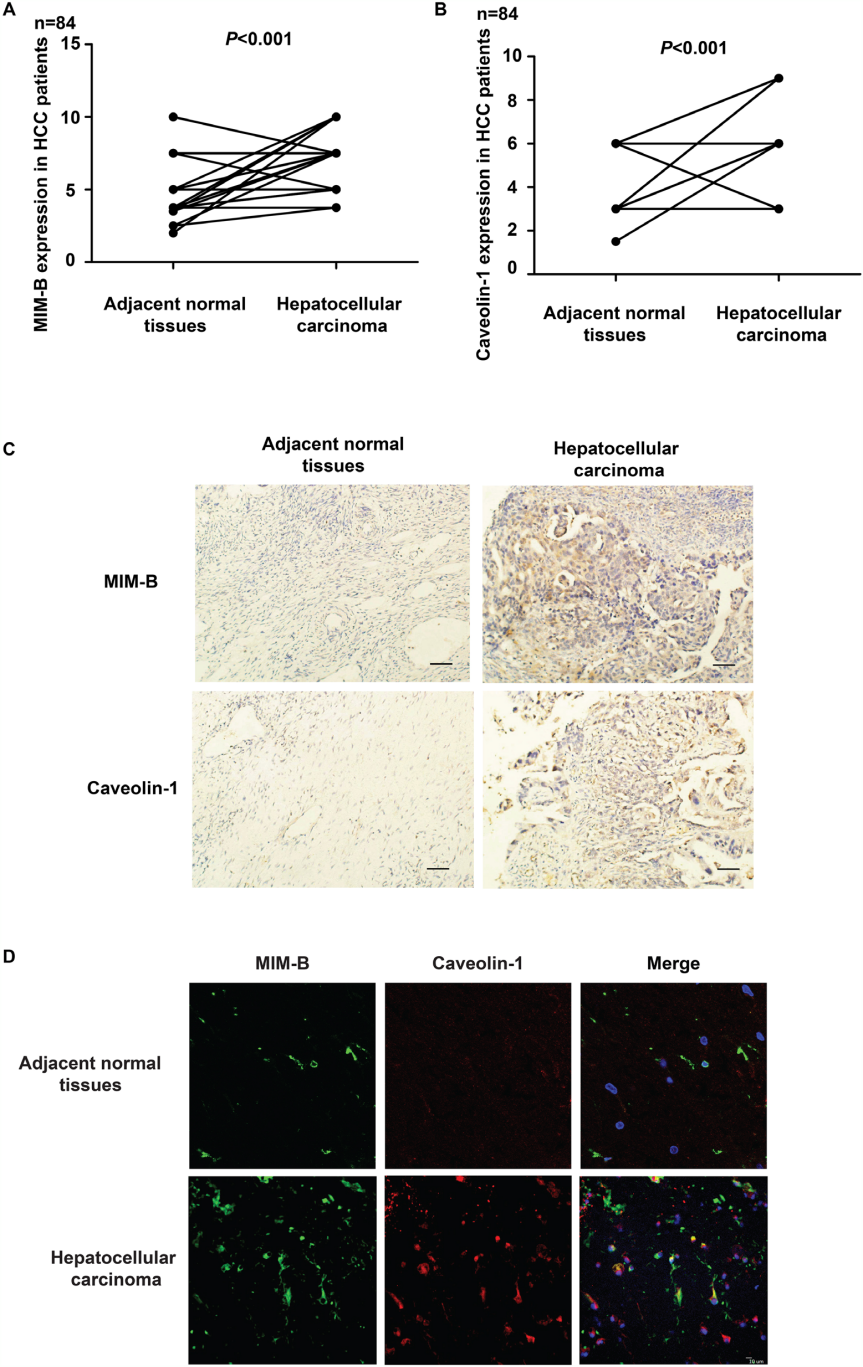
MIM-B and caveolin-1 are aberrantly expressed in HCC **(A)** IHC showing that the difference in MIM-B expression levels between HCC tumor tissues and matched adjacent normal tissues is statistically significant (*P* < 0.001). **(B)** IHC showing that the difference in caveolin-1 expression levels between HCC tumor tissues and matched adjacent normal tissues is statistically significant (*P* < 0.001). **(C)** Representative images showing typical MIM-B and caveolin-1 IHC staining patterns in HCC tumors and matched adjacent normal tissues (scale bar, 50 μm). N = No. of paired samples. **(D)** MIM-B and caveolin-1 were significantly elevated and co-localized in HCC tissue samples when compared with matched adjacent normal tissues (*P* < 0.001).

To elucidate the possible roles of MIM-B and caveolin-1 expression with in HCC tissues, we compared their expression levels in clinical HCC samples and paired adjacent normal tissues. We found that MIM-B and caveolin-1 were differentially expressed between tumor tissues and normal tissues (Figure 6A–6D). We noted elevated MIM-B expression in 49 of 84 patient samples and increased caveolin-1 expression in 53 of 84 patient samples (*P* < 0.001, Figure 6A and 6B). Consistent with the data obtained from IHC, we found that MIM-B and caveolin-1 were significantly elevated and co-localized in HCC tissue samples when compared with matched adjacent normal tissues (*P* < 0.001, Figure 6D). Taken together, these results demonstrated that MIM-B and caveolin-1 expression levels were higher in cancer tissues than in paired adjacent normal tissues and that MIM-B and caveolin-1 expression levels were correlated with HCC clinico-pathologic characteristics.

**Figure 7:**
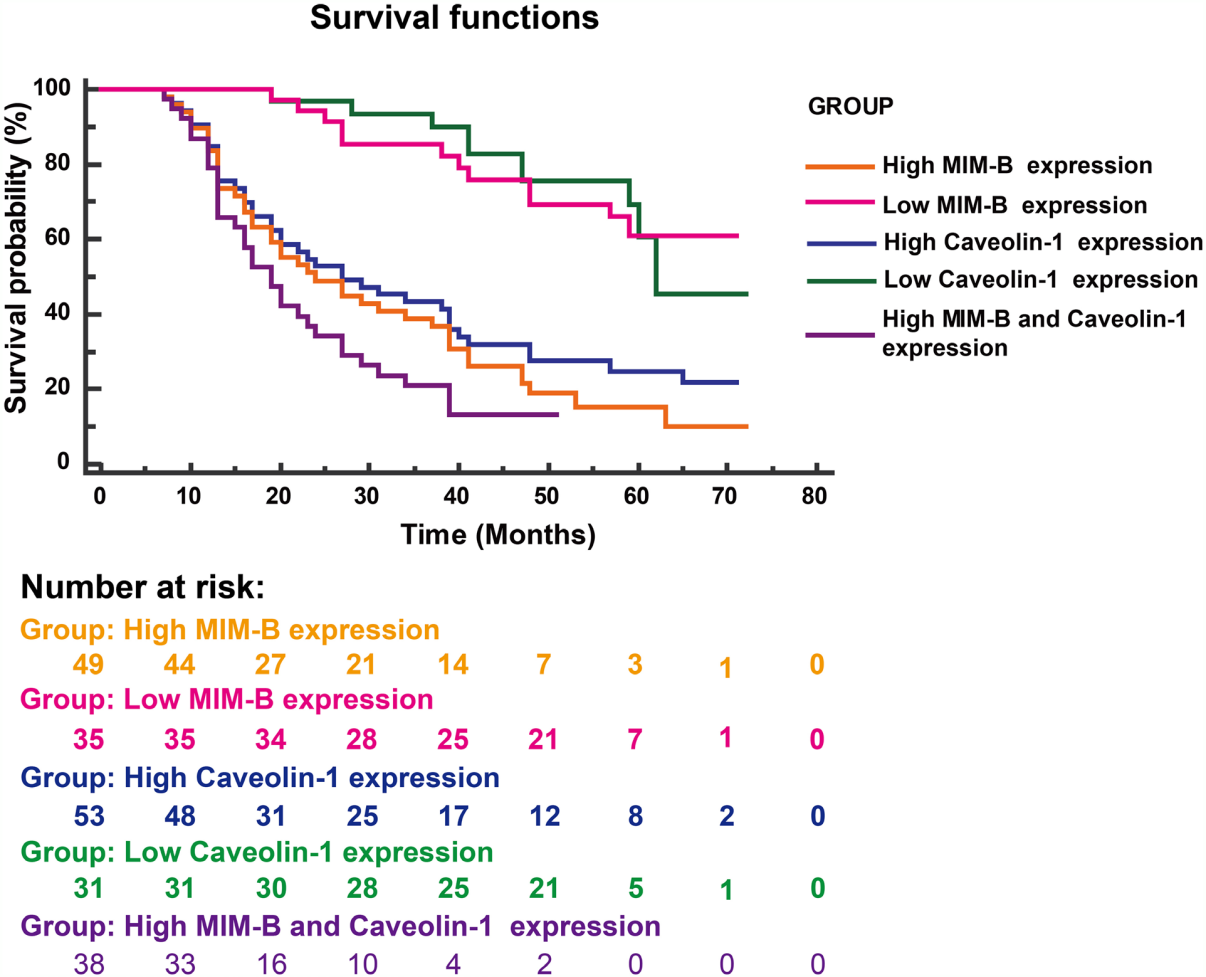
Kaplan-Meier survival plots for patients with different MIM-B and caveolin-1 expression levels Kaplan-Meier survival analysis (log-rank test) was applied for survival analysis. High MIM-B expression, n = 49; high caveolin-1 expression, n = 53; low MIM-B expression, n = 35; low caveolin-1 expression, n = 31; high MIM-B and caveolin-1 expression, n = 38. Patients with high MIM-B expression revealed poorer prognosis when compared with patients with low MIM-B expression (*P* < 0.0001); patients with high caveolin-1 expression revealed poorer prognosis when compared with patients with low caveolin-1 expression (*P* < 0.00001).

To confirm whether the combination of MIM-B and caveolin-1 can serve as a prognostic factor for HCC, we analyzed the overall survival of patients with different levels of MIM-B and caveolin-1 expression using the Kaplan-Meier method (log-rank test). We found that MIM-B and caveolin-1 expression levels were associated with overall survival (Figure 7). Patients with high MIM-B expression revealed poorer prognosis when compared with patients with low MIM-B expression (*P* < 0.0001, Figure 7); patients with high caveolin-1 expression revealed poorer prognosis when compared with patients with low caveolin-1 expression (*P* < 0.00001, Figure 7). The median survival durations in the low MIM-B and caveolin-1 expression groups were NA and 62 months respectively, while the mean survival durations in the high MIM-B and caveolin-1 expression groups were 24 and 27 months, respectively. Moreover, the mean survival duration in the high MIM-B + caveolin-1 expression group was 19 months (*P* < 0.01).

Collectively, these results demonstrated that MIM-B/caveolin-1 expression may be an independent prognostic factor for HCC, as well as a novel therapeutic target in the treatment of HCC.

## DISCUSSION

In this study, we found that MIM-B could co-localize and interact with caveolin-1 in HCC cells and that inhibiting MIM-B and caveolin-1 had an inhibition effect on EGFR pathway activity. Furthermore, we showed that altering MIM-B and caveolin-1 expression levels could affect MIM-B and caveolin-1 interactions and HCC cell invasion and migration. Through a series of experiments, we found that MIM-B and caveolin-1 expression levels were higher in cancer tissues than in paired adjacent normal tissues and that MIM-B and caveolin-1 expression levels were correlated with HCC clinico-pathologic characteristics.

In previous studies, MIM-B was found to act as an actin-monomer binding protein that sequestered actin monomers and induced Rac activation and lamellipodia formation [15, 21]. However, the mechanism through which MIM-B exerts these effects remains largely unknown. In this study, immunofluorescence assay indicated that caveolin-1 may be an interaction partner for MIM-B (Figure 1A). Furthermore, the results of the co-IP assay demonstrated that MIM-B could interact with caveolin-1 *in vitro* (Figure 1). These results showed that MIM-B facilitated cell migration by interacting with caveolin-1. Caveolin-1 and MIM-B interact to exert a variety of effects. For example, previous studies reported that caveolin-1-mediated survival downregulation in the presence of E-cadherin coincided with increases in apoptosis facilitated by reductions in beta-catenin-dependent transcription and that MIM-B co-localized with E-cadherin in MDCK cells [48, 49].

The EGFR pathway plays important roles in a variety of cancers by affecting cell growth, cell metastasis, cell cycle activity, and cell differentiation [36, 39]; however, the mechanism through which the pathway alters the above processes remains largely unknown. The migration and invasion assays demonstrated that inhibiting MIM-B and caveolin-1 expression inhibited EGFR pathway activity (Figure 3C). Thus, these experiments demonstrated a new means by which the EGFR pathway is regulated in HCC. However, the effects of MIM-B on EGFR pathway activity are often different in different cell types [42]. Our studies provided us with clues regarding the mechanisms underlying the effects of MIM-B and caveolin-1 in HCC and have also provided us with a theoretical basis for studying the clinical applicability of MIM-B/caveolin-1 interactions.

Although MIM-B was found to be a metastasis suppressor gene in bladder cancer, increasing amounts of evidence indicate that its role in cancer cells is a controversial subject [4, 15–17]. The transwell migration assays results demonstrated that downregulating MIM-B could significantly inhibit MHCC97H cell metastasis (Figure 5). The role of caveolin-1 in different cancer cells is also a controversial subject. We found that knocking down caveolin-1 in MHCC97H cells had the same effect as knocking down MIM-B (Figure 5). Our study demonstrated that MIM-B and caveolin-1 could promote MHCC97H cell migration. Consistent with this result, the results of subsequent experiments showed that cell migration ability of MHCC97H cells in which MIM-B and caveolin-1 expression was suppressed was significantly decreased compared with that of cells in which the two proteins were expressed (Figure 5). These results suggested that MIM-B and caveolin-1 play important and similar roles in regulating cell migration.

There are few biomarkers specific for solid tumors such as HCC. Several reports have documented that both MIM-B and caveolin-1 expression levels are significantly altered in various type of cancers and may thus be an independent prognostic factor for different cancers [50–54]. In this study, interestingly, we demonstrated that loss of MIM-B, caveolin-1 or both could inhibit MHCC97H cell migration (Figure 5). Furthermore, our study showed that MIM-B expression levels were elevated in 49 of 84 patient samples and that caveolin-1 levels were elevated in 53 of 84 patient samples (*P* < 0.01) (Figure 6A and 6B). All human HCC tissue samples were collected from patients with hepatitis B virus infection and no patients had T4 or IVB disease in this study. Tumor stage (T stage) means tumor size. MIM-B is not related with T stage, but related with TNM stage (*P* = 0.002), encapsulation (*P* = 0.005), vascular invasion (*P* = 0.000), which could be found in Table 1. Our results were consistent with the previous study [14], which demonstrated that MIM-B expression was significantly associated with early pathologic TNM stage group (*P* = 0.007), presence of tumor encapsulation (*P* = 0.034), and absence of venous infiltration (*P* = 0.038).

These results indicated that MIM-B/caveolin-1 expression may be an independent prognostic factor for HCC and may also be a novel therapeutic target in the treatment of HCC. However, these hypotheses warrant further study, and we are currently designing experiments involving HCC samples from patients to confirm them.

## MATERIALS AND METHODS

### Cells and reagents

The cell lines used herein were cultured in media, as recommended by the American Type Culture Collection (ATCC). The human HCC cell MHCC97H cell lines was purchased from the Liver Cancer Institute, Shanghai Medical College of Fudan University, and was cultured in DMEM supplemented with 10% FBS, 100 μg/ml penicillin, 0.1 mg/ml streptomycin and 2 mM L-glutamine at 37°C in a humidified atmosphere containing and 5% CO_2_. Puromycin (GM-040401-1) was purchased from Genomeditech (Shanghai, China), and hEGF (Sigma–Aldrich, St. Louis, MO, USA) was dissolved in a solution of phosphate buffered saline (PBS) (pH=7.4).

### Tissue specimens

This study was approved by the Research Ethics Committee of Shanghai Jiaotong University Affiliated Sixth People's Hospital. Human HCC tissue samples were collected from patients who underwent surgical resection in Shanghai Jiaotong University Affiliated Sixth People's Hospital and provided informed consent regarding the use of their samples in this study. The criteria used to determine whether patients were eligible for inclusion in this study were reported in a previous study [12]. In brief, the inclusion criteria for patients in this study were (a) pathologically proven HCC based on WHO criteria and UICC-TNM classification; (b) no anticancer treatment prior to hepatectomy; (c) patients with hepatitis B; (d) availability of frozen resected HCC tissues and follow-up data. The samples were collected from 2008 to 2012 and were evaluated until December 31, 2015. All specimens were evaluated and reclassified according to the current WHO and UICC-TNM classifications (Travis et al, 2004) (7th edition; Sobin et al, 2009). Fresh HCC tissues and paired adjacent normal tissues (≥ 2 cm away from tumor) were collected in the operating room immediately (≤ 15 min) after tissue removal and were snapping frozen in liquid nitrogen and stored at -80°C until use. Their histological types were confirmed using standard hematoxylin and eosin staining. A summary of the clinico-pathological characteristics of the samples is provided in Table 1.

### Plasmid construction, lentivirus production and cell infection

The MIM-B and caveolin-1 expression plasmids used herein were based on the pLVX-puro-EGFP lentiviral vector. The following MIM-B primers were used in this study: F: 5′- CTAGCTAGCATGGAGGCTGTGATTGAGAAGG-3′ and R: 5′- CGGGATCCCTAAGAAAAGCGAGGGGCTGAG-3′. The following caveolin-1 primers were used in this study: F: 5′-CTAGCTAGCATGTCTGGGGGCAAATACGTAG-3′ and R: 5′- CGGGATCCTTATATTTCTTTCTGCAAGTTGATGCGG-3′.

MIM-B and caveolin-1-specific small-hairpin RNA (shRNA) sequences, which were synthesized by General Biosystems (Anhui), were ligated into a pLKO.1 lentiviral vector. The MIM-B sequences were as follows: sense: 5′-GATCCCCGTAGATGCATTGTAGTAGT-3′, and antisense: 5′-ACTACTACAATGCATCTACGGGGATC-3′. The caveolin-1 sequences were as follows: sense: 5′-GATCCCCAACCAGAAGGGACACACAGTT-3′, and antisense: 5′-AACTGTGTGTCCCTTCTGGTTGGGGATC-3′.

The lentiviruses were produced as follow: shuttle vectors and helper plasmids were transfected into 293FT cells for 48 h with Lipofectamine 2000, according to the manufacturer's instructions (Invitrogen, USA). The cells were seeded in the appropriate plates for approximately 18 h before being transfected with the indicated plasmids.

We constructed MIM-B gene fragments lacking the sequences for the IMD or WH2 motifs and inserted these fragments into a pcDNA-3.1 plasmid. Specifically, the MIM-IMD.ko and MIM-WH2.ko fragments were amplified and inserted into the indicated plasmid, which contained BamHI/XhoI restriction sites. The plasmids were then transfected into the above cells for further research.

### RNA extraction and quantitative real-time PCR

Total RNA was extracted with TRIzol regent (Invitrogen), according to the manufacturer's instructions. One microgram of RNA was subsequently reverse transcribed into cDNA with a Takara PrimeScript RT Regent Kit (Takara), and the cDNA was amplified by real-time PCR on an Applied Biosystems 7500 (Applied Biosystems). GAPDH was used as a control, and the normalized expression levels of the target genes were expressed using arbitrary units. The following primers for MIM-B were used in the experiment: F: 5’-TGCAGAAGAAAGCAAA-3’ and R: 5’-GGAGAGCACTGTCCAACTGA-3’. The following primers for caveolin-1 were used in the experiment: F: 5’-TCTTTGGCATCCCGATGG-3’ and R: 5’-GTTGATGCGGACATTGCT-3’. The following primers for GAPDH were used in the experiment: F: 5’-GCACCGTCAAGGCTGAGAAC-3’ and R: 5’-ATGGTGGTGAAGACGCCAGT-3’.

### Western blotting

Western blot analysis was performed as described previously [55]. The following antibodies, which were supplied by Abcam (Cambridge, UK), were used in this experiment: anti-MIM-B (Abcam78161), anti-caveolin-1 (Abcam2910), anti-p-EGFR(Abcam52894), anti-EGFR (Abcam52894), anti-p-STAT3 (Abcam76315), anti-STAT3 (Abcam119352), anti-p-AKT (Abcam38449), anti-AKT (Abcam8805), anti-p-ERK (Abcam176660), and anti-ERK (Abcam54230). Anti-Actin (Rabbit, DH0251#), anti-GAPDH (Rabbit, DH0261#) and ECL for Western Blotting Substrate (DH0101) were also used in this experiment and were supplied by Donghuan Biotech Co., Ltd (Shanghai).

### Co-immunoprecipitation (Co-IP) assay

Cultured cells were washed twice with ice-cold PBS and then lysed in 500 μl of ice-cold RIPA lysis buffer (1% NP-40, 50 mM Tris-HCl (pH 7.4), 150 mM NaCl, 1 mM EDTA, 0.5 mM PMSF, 1 mM DTT and 1×Protein Inhibitor (Roche)). The whole-cell lysates were subsequently centrifuged at 12,000 rpm for 10 min at 4°C, after which the cell supernatant was subjected to immunoprecipitation with the appropriate primary antibody for 1 h at 4°C before being captured with protein A/G-coupled sepharose beads. After being washed 4 times with ice-cold lysis buffer, the beads were re-suspended in protein loading buffer and then subjected to western blot analysis.

### Immunofluorescence staining and confocal microscopy

After being washed with PBS, cultured MHCC97H cells were fixed with 4% PFA (in PBS) for 10 min before being permeabilized with PBS-0.5% Triton and blocked with PBS-5% inactivated goat serum for 1 h. The cells were then incubated in PBS containing 5% BSA and the appropriate primary antibodies for at least 2 h at room temperature, after which they were incubated with the appropriate IgGAlexafluorescent-conjugated secondary antibodies for 1 h. The nuclei were stained with DAPI for 5 min, and then the cells were mounted with cover slips after being washed with PBS. For protein expression and colocalization in HCC and the adjacent normal tissue samples, sections were incubated in a hydrogen peroxide solution (0.3%) for 1 hour at room temperature. The slides were then analyzed with a confocal microscope (Leica) using a 63×magnification lens.

### Cell migration and invasion

Cell migration and invasion assays were performed with a transwell apparatus (Millipore), according to the manufacturer's instructions. Briefly, after being serum starved for 24 h, the cells were seeded in the top chamber of the transwell apparatus, which was coated with collagen IV, and medium containing fibronectin was added to the bottom chamber of the apparatus. The cells were subsequently incubated for 48 h at 37°C and then fixed with 0.1% methanol for 10 min before being stained with hematoxylin. The non-migrating cells on top of each filter were removed with a cotton swab. The migrating cells were counted, and the results were expressed as the ratio of migrating or invading cells: control cells.

### Immunohistochemistry (IHC)

IHC was performed as previously described [56]. Anti-MIM-B antibodies (Abcam, abcam78161) were used to identify MIM-B, and anti-caveolin-1 antibodies (Abcam, abcam2910) were used to identify caveolin-1. MIM-B and caveolin-1 expression scores were calculated as the product of the cell staining percentage and cell staining intensity. The cell staining intensity was scored as follows: (none) = 0, (weak) = 1, (medium) = 2, and (strong) = 3, and the cell staining percentage was scored as follows: < 33% = 1, 33 - 66% = 2, and ≥ 66% = 3. The expression scores were calculated using the following equation: expression score = staining intensity × staining percentage. Scores ≥ 3 were considered “high” scores, while lower scores were considered “low” scores. MIM-B and caveolin-1 expression scores were determined independently by two researchers.

### Data analysis

Survival analysis was conducted using the Kaplan-Meier method (log-rank test). The data are presented as the mean ± standard deviation and are representative of at least three experiments. Differences between two groups were evaluated using Student's *t*-test, and all *P*-values were two-tailed. The symbol ^*^ denoted a significant difference (*P* < 0.05), while ^**^ denoted a very significant difference (*P* < 0.01).

## Abbreviations

HCC: Hepatocellular carcinoma
MIM-B: Missing in metastasis B
EGFR: Epidermal growth factor receptor
EGF: Epidermal growth factor
Co-IP: Co-immunoprecipitation
shRNA: Small hairpin RNA
shNC: Negative control shRNA
